# The Siderophore Transporters Sit1 and Sit2 Are Essential for Utilization of Ferrichrome-, Ferrioxamine- and Coprogen-Type Siderophores in *Aspergillus fumigatus*

**DOI:** 10.3390/jof7090768

**Published:** 2021-09-16

**Authors:** Mario Aguiar, Thomas Orasch, Matthias Misslinger, Anna-Maria Dietl, Fabio Gsaller, Hubertus Haas

**Affiliations:** Institute of Molecular Biology/Biocenter, Medical University of Innsbruck, A-6020 Innsbruck, Austria; mario.aguiar@i-med.ac.at (M.A.); thomas.orasch@hki-jena.de (T.O.); matthias.misslinger@i-med.ac.at (M.M.); anna-maria.dietl@i-med.ac.at (A.-M.D.); fabio.gsaller@i-med.ac.at (F.G.)

**Keywords:** fungi, molds, *Aspergillus fumigatus*, iron, siderophore, xenosiderophores, transporter

## Abstract

Siderophore-mediated acquisition of iron has been shown to be indispensable for the virulence of several fungal pathogens, the siderophore transporter Sit1 was found to mediate uptake of the novel antifungal drug VL-2397, and siderophores were shown to be useful as biomarkers as well as for imaging of fungal infections. However, siderophore uptake in filamentous fungi is poorly characterized. The opportunistic human pathogen *Aspergillus fumigatus* possesses five putative siderophore transporters. Here, we demonstrate that the siderophore transporters Sit1 and Sit2 have overlapping, as well as unique, substrate specificities. With respect to ferrichrome-type siderophores, the utilization of ferrirhodin and ferrirubin depended exclusively on Sit2, use of ferrichrome A depended mainly on Sit1, and utilization of ferrichrome, ferricrocin, and ferrichrysin was mediated by both transporters. Moreover, both Sit1 and Sit2 mediated use of the coprogen-type siderophores coprogen and coprogen B, while only Sit1 transported the bacterial ferrioxamine-type xenosiderophores ferrioxamines B, G, and E. Neither Sit1 nor Sit2 were important for the utilization of the endogenous siderophores fusarinine C and triacetylfusarinine C. Furthermore, *A. fumigatus* was found to lack utilization of the xenosiderophores schizokinen, basidiochrome, rhizoferrin, ornibactin, rhodotorulic acid, and enterobactin. Taken together, this study characterized siderophore use by *A. fumigatus* and substrate characteristics of Sit1 and Sit2.

## 1. Introduction

Iron is an essential element for almost all organisms since it serves as a cofactor of numerous cellular processes. In excess, however, this metal can be highly toxic by promoting the production of reactive oxygen species [1]. Consequently, iron homeostatic mechanisms are essential to balance the uptake, storage, and use of this metal. Despite its high abundance in the Earth’s crust, the bioavailability of iron is low due to its oxidation by atmospheric oxygen and the formation of sparingly soluble ferric (Fe^3+^) hydroxides by atmospheric oxygen. Therefore, microorganisms evolved different strategies for iron acquisition. The mold *Aspergillus fumigatus* is the most common and life-threatening opportunistic airborne fungal pathogen in humans [2]. In addition to non-invasive forms of aspergillosis, patients with a compromised immune system are at high risk of developing invasive aspergillosis. The limitations in diagnosis and therapy result in high mortality rates of invasive aspergillosis [2]. *A. fumigatus* employs two high-affinity iron acquisition mechanisms, reductive iron assimilation (RIA) and siderophore-mediated iron acquisition, as well as low-affinity ferrous (Fe^2+^) iron uptake [3]. RIA involves the extracellular reduction of Fe^3+^ by membrane-bound metalloreductases such as FreB into Fe^2+^ followed by reoxidation and cellular uptake of iron by a protein complex consisting of the ferroxidase FetC and the iron permease FtrA [4,5]. Siderophores are low molecular mass ferric-iron chelators, which fall into different structural classes termed hydroxamates, catecholates, carboxylates, phenolates, and mixed-classes [6]. Within these classes, there is huge structural variety, which most likely reflects the role of siderophore-mediated iron acquisition in microbial competition [3,7]. Within hydroxamates, rhodotorulic acid, ferrioxamine-, fusarinine-, coprogen-, and ferrichrome-type siderophores are discriminated. Ascomycetes such as *A. fumigatus* produce exclusively hydroxamate-class siderophores. Table 1 summarizes the siderophores used or discussed in this work. *A. fumigatus* secretes two fusarinine-type siderophores, triacetylfusarinine C (TAFC) and fusarinine C, to solubilize and sequester environmental iron and employs two ferrichrome-type siderophores for intracellular handling of iron, hyphal ferricrocin and conidial hydroxyferricrocin [8]. The biosynthesis of hydroxamate-class siderophores involves several enzymes and cellular compartments with the first dedicated enzymatic step being hydroxylation of ornithine [8,9]; i.e., inactivation of the ornithine hydroxylase SidA blocks the biosynthesis of all siderophores in *A. fumigatus*. After the secretion and chelation of iron, the siderophore-iron complexes are taken up by specific transporters, which belong to the “Siderophore-Iron-Transporter” (SIT)-subfamily of the major facilitator protein superfamily [10]. The presence of SITs is confined to the fungal kingdom and even species that lack the production of siderophores, such as *Saccharomyces cerevisiae*, *Candida albicans*, *Candida glabrata,* or *Cryptococcus neoformans*, possess SITs for the uptake of xenosiderophores, i.e., non-self-produced siderophores [8]. SITs are commonly composed of 400–600 amino acids that fold into 12 to 14 transmembrane helices [11] and most likely act as proton symporters by using the ionic (H^+^) gradient across the membrane as the source of energy [4,12,13]. The substrate specificity of SITs is best characterized in the siderophore non-producer *S. cerevisiae,* which possesses four SITs [14]. However, phylogenetic analysis revealed that all *S. cerevisiae* SITs are more similar to each other than they are to SITs from other fungal species [10], which indicates that these transporters arose after the split from the other species. Consequently, the substrate specificity of *A. fumigatus* SITs cannot be predicted on the basis of sequence similarity to *S. cerevisiae* SITs. *A. fumigatus* possesses five potential SITs, termed MirB, MirC, MirD, Sit1 and Sit2, which are transcriptionally repressed by iron indicating a role in iron homeostasis [15]. MirB was found to transport the endogenous secreted siderophore TAFC [16,17], Sit1 was found to transport the xenosiderophores ferrichrome and ferrioxamine B [18] and Sit2 was found to also transport ferrichrome [18]. Ferrichrome is produced by several fungal species and ferrioxamines are produced by bacterial species such as streptomyces and *Erwinia* spp. [19,20]. The substrate specificities of MirC and MirD remain to be elucidated, whereby MirC has been suggested to participate in ferricrocin biosynthesis [21]. Furthermore, *A. fumigatus* has been shown to take up ferrioxamine E and coprogen but the respective SITs have not been characterized [22].

Siderophore-mediated iron assimilation is found in fungi and bacteria. Fungal siderophore biosynthesis raised considerable interest because it was found to be essential for the virulence of several animal- and plant-pathogenic fungal species including *A. fumigatus* [3]. Moreover, siderophores were found to have great potential as biomarkers for fungal infections in humans [23,24,25], for in vivo imaging of fungal infections [26,27,28], and for therapeutic applications via coupling of toxic compounds as a Trojan horse approach [29]. Furthermore, *A. fumigatus* Sit1 was found to mediate uptake of the novel antifungal drug VL-2397, also termed ASP2397 [30]. Therefore, the exact substrate specificities of the *A. fumigatus* SITs are not only of scientific but also of translational interest as SITs might serve as a target for therapy and/or diagnosis of fungal infections. The analysis of substrate specificities of Sit1 and Sit2 was previously characterized by heterologous expression in *S. cerevisiae* and short-term uptake studies in *A. fumigatus* mutants lacking Sit1 and/or Sit2 focusing exclusively on ferrichrome and ferrioxamine B [18]. To comprehensively characterize siderophore utilization of *A. fumigatus* and particularly of Sit1 and Sit2, we applied an alternative approach. We generated mutants lacking Sit1 and/or Sit2 in a genetic background avoiding interference with endogenous siderophores and RIA to enable characterization of siderophore uptake by growth studies. This strategy allows analysis of a variety of siderophores without radiolabeling and to identify exclusivity of substrate specificity. Taken together, this study revealed that *A. fumigatus* is able to utilize a wide spectrum of siderophores with different efficiency and identified several siderophores that cannot be used by this mold. Moreover, the overlapping and specific substrate specificities of Sit1 and Sit2 characterized here provide structural information of the respective substrates. Furthermore, phylogenetic analysis revealed insights into (i) the evolutionary conservation of Sit1 and Sit2, (ii) the relationship of Sit1 and Sit2, and (iii) the value of phylogenetic analysis for substrate prediction of SITs.

**Table 1:** Siderophores used or discussed in this study [6].

Class	Type	Example ^1^	Exemplary Producer ^2^
Hydroxymates	fusarinines	fusarinine C	*A. fumigatus, A. nidulans*
		triacetylfusarinine C	*A. fumigatus, A. nidulans*
	coprogens	coprogen	*Penicillium chrysogenum*
		coprogen B	*Neurospora crassa*
	ferrichromes	ferrichrome	*A. nidulans, Ustilago maydis*
		ferrichrome A	*Ustilago maydis*
		ferricrocin hydroxyferricrocin	*A. fumigatus, A. nidulans* *A. fumigatus*
		ferrichrysin	*A. terreus, A. flavus*
		ferrirubin	*A. ochraceous*
		ferrirhodin	*Ceratobasidium globisporum*
		VL-2397	*Acremonium persicinium*
	ferrioxamines	ferrioxamine B	*Streptomyces pilosus*
		ferrioxamine E	*Streptomyces olivaceus*
		ferrioxamine G	*Erwinia amylovora*
	rhodotorulic acid	rhodotorulic acid	*Rhodotorula glutinis*
	trishydroxamate	basidiochrome	*Rhizoctonia mucoroides*
Carboxylates	hydroxycarboxylate	rhizoferrin	*Rhizopus delemar, Francisella tularensis*
Mixed types	hydroxamate/ hydroxycarboxylate	ornibactin schizokinen	*Burkholderia cepacia* *Bacillus megaterium*
		pyoverdine ^3^	*Pseudomonas aeruginosa*
Catecholates		enterobactin	*Escherichia coli*

^1^ Siderophores produced by *A. fumigatus* are shown in blue, VL-2397 is shown in red, siderophores not utilized by *A. fumigatus* are shown in green (this study) and other siderophores are in black; ^2^ *A.*: *Aspergillus*; ^3^ pyoverdine data are from [31].

## 2. Materials and Methods

### 2.1. Growth Conditions

For spore production and plate growth assays, *A. fumigatus* strains were grown on *Aspergillus* minimal medium (AMM) [32] containing 1% (*w*/*v*) glucose and 20 mM glutamine as carbon and nitrogen sources, respectively. For iron-replete conditions, FeSO_4_ was added to a final concentration of 0.03 mM; for iron starvation, iron addition was omitted. For spore production of *A. fumigatus* Δ*sidA*Δ*ftrA* strains, an iron concentration of 5 mM was used. For point-inoculation on plates, 10^4^ spores were used; for inoculation of liquid media, 10^6^ spores per mL of medium were used; incubation of solid and liquid cultures was performed at 37 °C. Supplementation with siderophores was performed during pouring the plates at 65 °C.

### 2.2. A. fumigatus Mutant Strains Generation

All studies were carried out in *A. fumigatus* strain AfS77, a derivative of *A. fumigatus* ATCC46645 lacking non-homologous recombination (*akuA*::*loxP*), which facilitates genetic manipulation [33,34]. For the generation of the selection marker-free ∆*sidA*/∆*ftrA A. fumigatus* mutant strain, *sidA* (AFUA_2G07680) and *ftrA* (AFUA_5G03800) were replaced in a PEG-mediated transformation approach by a self-excising hygromycin resistance cassette (*hph*) containing the β-rec/six site-specific recombination system under the control of the xylose-inducible promoter [33,35]. Therefore, 1.0 kb 5′- and 3′-non-coding regions (NCR) of *sidA* and *ftrA* were amplified by PCR using the primer pairs TO16/TO17 (*sidA* 5′-NCR), TO18/TO19 (*sidA* 3′-NCR), TO20/TO21 (*ftrA* 5′-NCR) and TO22/TO23 (*ftrA* 3′-NCR). The self-excising *hph* resistance cassette was excised from plasmid pSK529 by digestion with the restriction enzyme *Fsp*I. This fragment, together with the respective NCR fragments, were assembled by a NEBuilder reaction (NEBuilder© HiFi DNA Assembly, New England Biolabs, Ipswich, MA, USA) in a pUC19L (Thermo Fisher, Waltham, MA, USA) backbone, resulting in the plasmids p∆*sidA*-rec and p∆*ftrA*-rec. These plasmids were used as templates to amplify the deletion constructs with a size of 6.8 kb for transformation by PCR using the primer pairs TO56/TO57 for *sidA* or TO60/61 for *ftrA*, respectively. Selection for *sidA* deletion transformants was performed on AMM containing 0.2 mg/mL hygromycin B (Calbiochem©, San Diego, CA, USA), the resistance cassette was excised from Δ*sidA* mutants by cultivation on AMM containing 1% xylose. Subsequently, *ftrA* was deleted with the same strategy. Correct genotypes were confirmed by Southern blot analysis. The resulting Δ*sidA*Δ*ftrA* strain was then used as the background strain for further genetic manipulations. For all other genetic manipulations, non-self-excising selection markers were employed.

For *sit1* (AFUA_7G06060) deletion in the *∆sidA∆ftrA* background, genomic DNA from a previously generated ∆*sit1* strain [30] was used to amplify the *sit1* deletion cassette by using oligonucleotides TO102/TO105. This fragment contains 5’- and 3´-NCR of *sit1* and an interjacent *hph* cassette.

For C-terminal Venus-tagging of Sit1, plasmid pMA04 was generated by including the *sit1* encoding sequence followed by the GFP-derivative mVenus-encoding gene amplified with oligonucleotides MA07/MA12 from plasmid pMMHL69 [30], an *hph* cassette amplified with MA13/MA14 from pMMHL69 and a fragment containing the 3′ -NCR of *sit1* amplified with oligonucleotides MA15/MA16 from genomic DNA. For N-terminal Venus-tagging of Sit1, the plasmid pMA05 was generated including *sit1* 5′-NCR amplified with MA07/MA17 from genomic DNA, a Venus-encoding fragment with MA18/MA19 from pMMHL69, the *sit1* region with MA12/MA20 from genomic DNA, an *hph* cassette with MA13/MA14 from pMMHL69 and the 3′ -NCR of *sit1* with oligonucleotides MA15/16 from genomic DNA. Fragments for pMA04 or pMA05, respectively, were assembled by a NEBuilder reaction (NEBuilder© HiFi DNA Assembly, New England Biolabs) in a pUC19L (Thermo Fisher) backbone. Fragments amplified from pMA04 or pMA05 with primers MA20/MA16 and MA07/MA16, respectively, were used in the *A. fumigatus* transformation rounds into wild-type.

For deletion of *sit2* (AFU_7G04730) the plasmid pMA01 containing the Δ*sit2* deletion cassette was designed with a 5′-NCR of *sit2*, a pyrithiamine resistance cassette (*ptrA*) and a 3′-NCR of *sit2*, individually amplified by PCR with oligonucleotides MA01/02, MA03/04 and MA05/06, respectively, using genomic DNA as a template for the NCRs and the plasmid pSK275 [34] for *ptrA*. Amplified fragments were assembled by a NEBuilder reaction (NEBuilder© HiFi DNA Assembly, New England Biolabs) in a pUC19L (Thermo Fisher) backbone. For transformation of *A. fumigatus*, the deletion construct was PCR-amplified from plasmid pMA01 with primers MA01/MA06.

For the complementation of ∆*sit1* and ∆*sit2* mutants in ∆*sidA*∆*ftrA* background, *sit1* and *sit2* genes including 1.5 kb 5´- and 3´-NCRs were integrated into the *fcyB* locus, which allows selection for 5-flucytosine resistance without the need of another selection marker [36]. Therefore, *sit1* and *sit2* genes were PCR-amplified from genomic DNA using primers MA53/54 and MA55/56, respectively. The resulted fragments were then assembled in a NEBuilder reaction (NEBuilder© HiFi DNA Assembly, New England Biolabs) into a pUC19L-*fcyB* vector containing the 5′ and 3′ flanking regions of *fcyB* locus [36]. The generated plasmids were linearized by *NotI*-digestion and the resulting fragments were used for transformation in *A. fumigatus*. The transformation of *A. fumigatus* AfS77 was performed according to Tilburn et al. 1983 [37]. Selection of transformants was carried on minimal medium plates with 0.2 mg/mL hygromycin B, 0.1 µg/mL pyrithiamine (Sigma©, Tokyo, Japan), or 10 µg/mL flucytosine (TCI©, Eschborn, Germany). Correct genetic manipulations were proven by Southern blot analysis and growth assays (Supplementary Figures S1–S3). Fungal strains and primers used in this study are listed in Supplementary Tables S1–S3.

### 2.3. Siderophores

Ferrichrome and ferrioxamine B were purchased from Sigma^®^ (F8014, Burlington, MA, USA); rhodotorulic acid and basidiochrome were gifts from Günther Winkelmann and Kurt Haselwandter, respectively. All other siderophores were purchased from EMC Microcollections, Germany. Triacetylfusarinine C, fusarinine C, and ferricrocin were produced and isolated *in-house* from iron-starved *A. fumigatus* liquid cultures as described previously [38,39].

### 2.4. Fluorescence Microscopy

For microscopy, spores of fluorescent-tagged fungal strains were grown in coverslips with AMM at 37 °C, under iron starvation or iron sufficiency. Mycelia were observed with a Zeiss Axioplan fluorescence microscope (Oberkochen, Germany) equipped with an Axiocam 503 mono microscope camera (Oberkochen, Germany) and excitation/emission filters at 428/536 nm for mVenus detection. Image processing and editing were made with ZEN 2 (Blue Edition) microscope software, Adobe Photoshop CS6 (v.13), and Microsoft Power Point (v.16).

### 2.5. Bioinformatics

Protein sequences of respective membrane transporters were obtained from FungiDB, the *Saccharomyces* Genome Database [40], the *Candida* Genome Database [41], and NCBI Database [42]. Multiple alignments were performed with the Geneious Prime (2021, v2.2) [43] algorithm. The phylogenetic tree was constructed from the multiple protein sequence alignment using the neighbor-joining method based on 100 replicates, also in Geneious Prime. The GenBank protein accession number follows each sequence name. Protter was used for domain organization prediction [44].

## 3. Results

### 3.1. Characterization of Siderophore Uptake in A. fumigatus Mutants Lacking Both RIA and Siderophore Biosynthesis in Combination with Deficiency in Sit1 and/or Sit2

**Figure 1:**
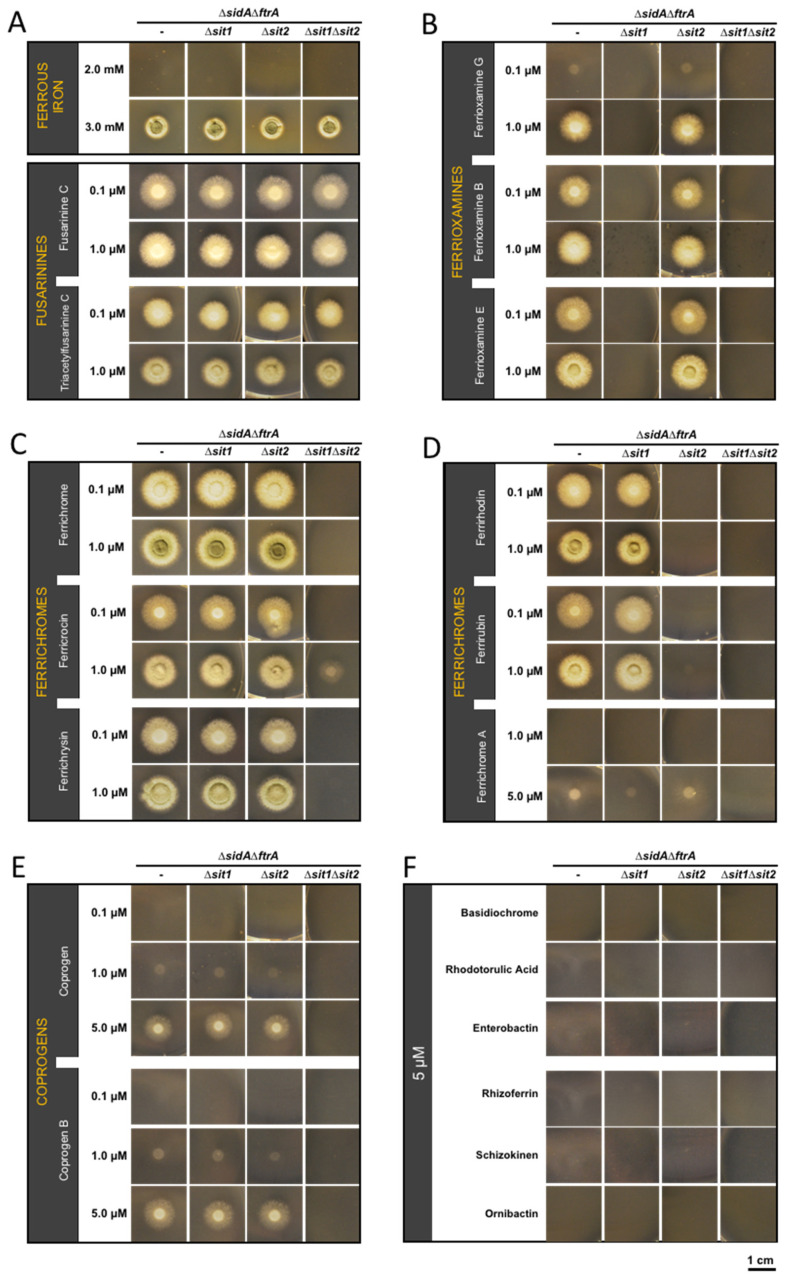
*A. fumigatus* Sit1 and Sit2 mediate utilization of ferrioxamine-, ferrichrome-, and coprogen-type siderophores. To analyze the role of Sit1 and Sit2 in the utilization of endogenous and exogenous siderophores, 1 × 10^4^ conidia of the mutant strains were point-inoculated on solid minimal medium supplemented with different concentrations of Fe^2+^ or ferric siderophores: (**A**) Fe^2+^ and fusarinine-type siderophores; (**B**) ferrioxamine-type siderophores; (**C**,**D**) ferrichrome-type siderophores; (**E**) coprogen-type siderophores; and (**F**) other siderophore-types. The plates were incubated at 37 °C for 48 h.

Analysis of the uptake of exogenously added siderophores in *A. fumigatus* is hampered by interference with the endogenous high-affinity iron acquisition systems RIA and endogenous siderophore production. To avoid this and to allow the characterization of siderophore uptake in *A. fumigatus* by simple growth studies, a selection marker-free mutant lacking siderophore biosynthesis (∆*sidA,* AFUA_2G07680) and reductive iron assimilation (∆*ftrA,* AFUA_5G03800) was generated using an excisable hygromycin (*hph*) selection marker [33]. A previously described *A. fumigatus* ∆*sidA*∆*ftrA* mutant strain [45] was less suitable for further molecular manipulation as it carries *hph*. As the previously described mutant, the new ∆*sidA*∆*ftrA* double mutant is able to grow only in the presence of ≥3 mM Fe^2+^ concentrations via the low-affinity iron uptake system or when supplemented with low concentrations (µM) of utilizable siderophores via high-affinity uptake by SITs (Figure 1). In order to characterize the substrate specificity of Sit1 (AFUA_7G06060) and Sit2 (AFUA_7G04730) we generated mutants lacking either Sit1 (∆*sidA*∆*ftrA*∆*sit1*) or Sit2 (∆*sidA*∆*ftrA*∆*sit2*) or both SITs (∆*sidA*∆*ftrA*∆*sit1*∆*sit2*) and assayed their growth on solid media supplemented with iron or siderophores (Figure 1). Supplementation with 0.1 µM fusarinine C or TAFC rescued growth of ∆*sit1*∆*sit2* and this was not affected by inactivation of Sit1 and/or Sit2 (Figure 1A), which indicates that neither Sit1 nor Sit2 play major roles in the transport of the endogenously secreted siderophores. In contrast, mutants lacking Sit1, or both Sit1 and Sit2, were unable to utilize ferrioxamine G, B, and E (Figure 1B) which suggests that Sit1 is the sole transporter for ferrioxamine-type siderophores. Notably, the three ferrioxamines displayed different growth promotion efficacy in the order ferrioxamine E > B > G (Figure 1B). Recently, two chemically modified ferrioxamine B derivatives, in which the terminal amino group was either acetylated or succinylated, have been reported to be utilized by *A. fumigatus* [28]. Similar to the other ferrioxamines, these derivatives required exclusively Sit1 for utilization (Figure S4 in Supplementary Materials). The simultaneous inactivation of Sit1 and Sit2, but not individual inactivation of either of these SITs, blocked the utilization of the ferrichrome-type siderophores ferrichrome and ferrichrysin and, furthermore, largely decreased the utilization of ferricrocin (Figure 1C). These results indicate that both Sit1 and Sit2 accept these three siderophores and that they are the exclusive transporters for ferrichrome and ferrichrysin, while ferricrocin appears to be transported to a low degree independent of Sit1 and Sit2. Mutants lacking Sit2, or both Sit1 and Sit2, were unable to utilize the ferrichrome-type siderophores ferrirhodin and ferrirubin (Figure 1D), which suggests that Sit2 is the sole transporter for these siderophores. The simultaneous inactivation of Sit1 and Sit2 blocked the utilization of the ferrichrome-type siderophore ferrichrome A, while individual inactivation of Sit2 but not Sit1 decreased the utilization of this siderophore (Figure 1D), which indicates that both SITs accept ferrichrome A but that Sit1 is the major transporter for this siderophore. Furthermore, the simultaneous inactivation of Sit1 and Sit2, but not individual inactivation of either of these SITs, blocked utilization of the coprogen and coprogen B (Figure 1E), which indicates that both Sit1 and Sit2 accept these two siderophores and that they are the exclusive transporters for these coprogen-type siderophores. Notably, these studies also revealed that utilization of ferrichrome A, coprogen, and coprogen B by *A. fumigatus* is poorer compared to the other siderophores as even higher concentrations (5 µM) still led to lower growth promotion (Figure 1D,E). Moreover, these growth assays revealed that *A. fumigatus* is not able to utilize the hydroxamate-class siderophores basidiochrome and rhodotorulic acid, the catecholate-class siderophore enterobactin, the carboxylate-class siderophore rhizoferrin, and the mixed-class siderophores ornibactin and schizokinen (Figure 1F). These data are summarized in Table 1.

To confirm gene deletion-specific effects, the ∆*sidA*∆*ftrA*∆*sit1*∆*sit2* mutant strain was complemented with either a functional *sit1* or a *sit2* gene copy by integration at the *fcyB* locus, yielding strains ∆*sidA*∆*ftrA*∆*sit2sit1^c^* and ∆*sidA*∆*ftrA*∆*sit1sit2^c^*. The growth pattern of these mutant strains on selected siderophores was identical to the respective single mutants, ∆*sidA*∆*ftrA*∆*sit2* and ∆*sidA*∆*ftrA*∆*sit1* (Figure 1 and Figure S5 in Supplementary Materials), which proves that the observed phenotypes are indeed caused by the specific gene deletion.

### 3.2. Sit1 Is Localized in the Plasma Membrane and Its Production Is Induced by Iron Starvation

**Figure 2:**
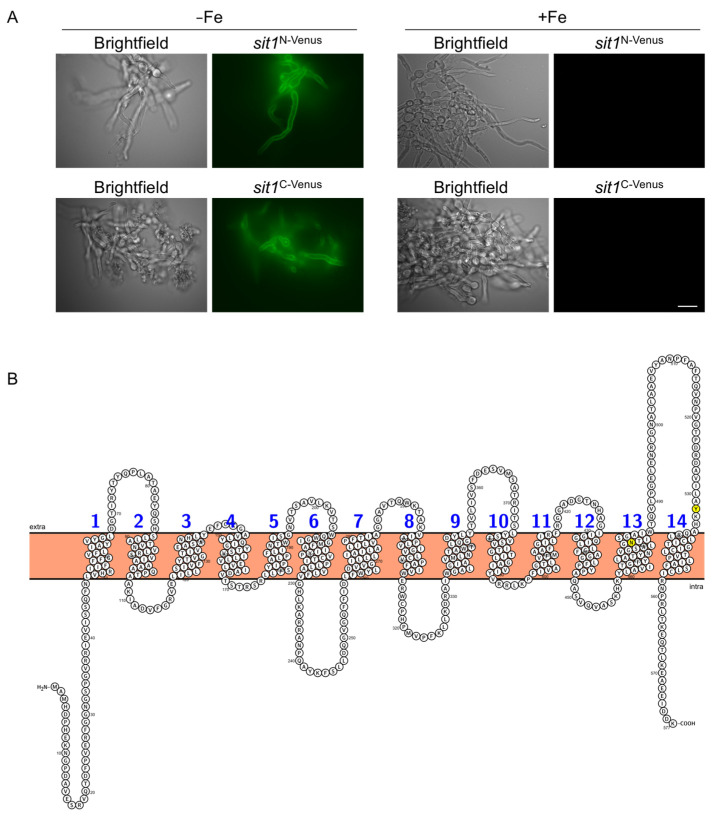
Sit1 is localized in the plasma membrane and its production is induced by iron starvation. (**A**) Epifluorescence microscopy of *A. fumigatus sit1*^N-Venus^ and *sit1*^C-Venus^ strains was performed as described in Materials and Methods; scale bar is 20 µm. (**B**) Schematic illustration of the Sit1 membrane topology according to Protter [44]. The point mutations rendering *A. fumigatus* resistant to VL-2397, N479K [46], and impairing Sit1 activity in *C. glabrata*, Y575A [47], are highlighted in yellow.

The expression of the genes encoding the five SITs has previously been shown to be repressed by iron through the iron regulatory transcription factor SreA [15]. To exemplarily analyze the protein localization and regulation of expression at the protein level, Sit1 was tagged N-terminally (Sit1^N-Venus^) and alternatively C-terminally (Sit1^C-Venus^) with the yellow fluorescence protein derivative Venus with expression of the tagged *sit1* alleles under control of the endogenous promoter as described in Materials and Methods. In agreement with the reported transcriptional regulation, epifluorescence microscopy demonstrated that the production of both Sit1^N-Venus^ and Sit1^C-Venus^ was repressed by iron (Figure 2A). Moreover, these data also confirmed the localization of both Venus-tagged Sit1 versions at the plasma membrane, as expected for a siderophore importer. According to the domain organization prediction using Protter [44], Sit1 has 14 predicted transmembrane regions (Figure 2B). The functionality of the Venus-tagged versions was confirmed by VL-2397 susceptibility testing as Sit1 mediates uptake of this antifungal drug [30]. Remarkably, particularly N- but also C-terminal Venus-tagging of Sit1 increased VL-2397 susceptibility (Table 2).

### 3.3. A. fumigatus Siderophore Transporters Belong to Different Subclades

**Figure 3:**
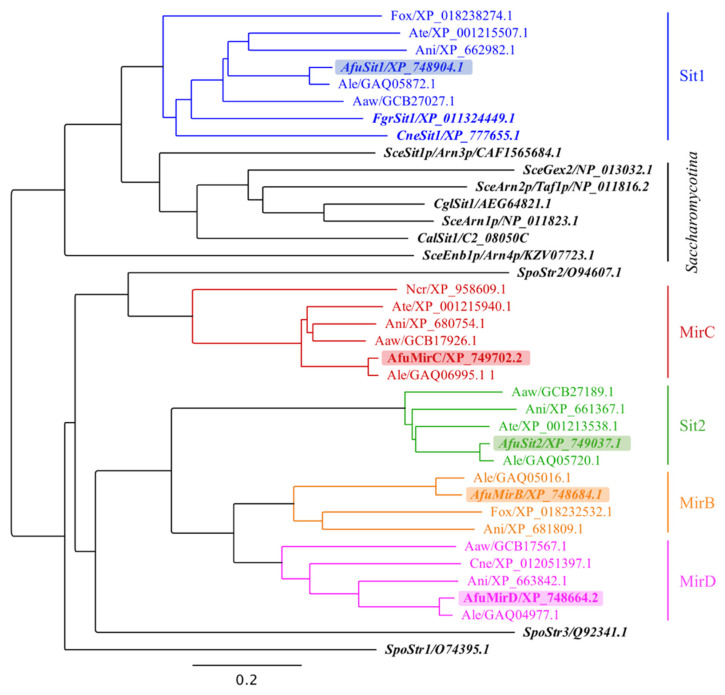
Phylogenetic analysis of selected SITs. Phylogenetic analysis was conducted with 38 SITs from *Aspergillus fumigatus* (Afu), *Aspergillus nidulans* (Ani), *Aspergillus lentulus* (Ale), *Aspergillus awamori* (Aaw), *Aspergillus terreus* (Ate), *Fusarium oxysporum* (Fox), *Fusarium graminearum* (Fgr), *Schizosaccharomyces pombe* (Spo)*, Saccharomyces cerevisiae* (Sce), *Neurospora crassa* (Ncr)*, Cryptococcus neoformans* (Cne), *Candida albicans* (Cal) and *Candida glabrata* (Cgl). Protein sequences alignment and neighbor-joining tree building were performed using the software Geneious Prime 2021.2.2 [43] (Scale bar is the percentage of genetic variation). SITs with identified substrates are in italics, SITs from *A. fumigatus* are shaded in different colors. The underlying multiple alignments for the corresponded sequences is displayed in Figure S7.

Phylogenetic analysis of 38 SITs from 12 fungal species including all SITs with identified substrates demonstrated that the five SITs of *A. fumigatus* belong to different subclades (Figure 3) [43]. Remarkably, despite overlapping substrate specificities, Sit1 and Sit2 are only distantly related.

## 4. Discussion

Table 3 summarizes the SITs that have previously been identified to transport ferrichrome- and/or ferrioxamine-type siderophores. In most cases, only a few substrates have been analyzed and substrate specificity of the SITs from the siderophore-producing species *Schizosaccharomyces pombe, Fusarium graminearum* and *A. fumigatus* was examined only by heterologous expression in *S. cerevisiae* and/or short-term uptake studies with radiolabeled siderophores. Consequently, most previous studies did not provide an in-depth description of substrate specificity. Moreover, these studies included only little data about siderophore producers (*S. pombe, F. graminearum and A. fumigatus*), as the experimental set-up is difficult due to the interference with endogenous siderophores.

**Figure 4:**
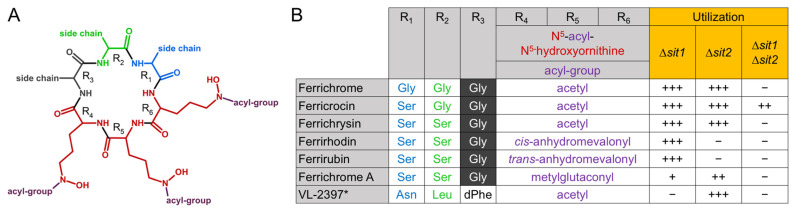
Comparison of the composition of different ferrichrome-type siderophores and their impact on recognition by *A. fumigatus* Sit1 and Sit2. (**A**) Schematic view of ferrichrome-type siderophores, which are cyclic hexapeptides with positions R_1_–R_6_. (**B**) Comparison of the ferrichrome-type siderophores used in this study with respect to composition and utilization. These ferrichrome-types differ in the N^5^-acyl groups present in positions R_4_–R_6_ and the amino acid residues present in positions R_1_–R_3_, whereby glycine (shaded in black) is present in position R_3_ in all these ferrichrome-types except VL-2397. Degree of utilization is marked by +++ high, ++ weak, + very weak and − no utilization. * Utilization of VL-2397 is taken from [30].

Here we characterized the substrate specificities of two transporters, Sit1 and Sit2, of the siderophore-producer *A. fumigatus*. To exclude interference with endogenous siderophore production and to allow maximal sensitivity, substrate specificity was analyzed by growth assays using mutants lacking Sit1 and/or Sit2 in a genetic *A. fumigatus* background lacking siderophore biosynthesis and RIA to avoid interference with endogenous siderophores and SIT-independent high-affinity uptake of iron from the supplemented ferric siderophores. These studies revealed that Sit1 is the sole transporter of *A. fumigatus* for ferrioxamine-type siderophores including cyclic ferrioxamine E as well as linear ferrioxamine G and ferrioxamine B and previously described ferrioxamine B derivatives, of which the terminal amino group was acetylated or succinylated [28]. In conclusion, recognition of ferrioxamines by Sit1 does not involve the termini of linear ferrioxamines, and modification of the termini does not block their uptake. These findings are possibly helpful for generating conjugates of ferrioxamines with toxic molecules to design novel antifungal drugs with microbial uptake specificity in a Trojan horse approach [26,53]. In this respect, it is noteworthy that SITs are fungal-specific transporters [10]; ferrioxamines are also used by bacteria but the transporters are of different types [54,55] and might have different characteristics for conjugates. The growth assays also indicated that efficacy of ferrioxamine utilization shows differences with ferrioxamine E > ferrioxamine B and ferrioxamine G. In line with this, uptake of ferrioxamine B was recently shown to display decreased efficacy compared to ferrioxamine E due to protonation of the terminal ferrioxamine B amino group particularly in acidic pH, while ferrioxamine E is a cyclic uncharged molecule [28]. Ferrioxamine G has not only a terminal amino group like ferrioxamine B but additionally a terminal carboxyl group [6], which might further decrease uptake efficacy due to being charged via deprotonation. Furthermore, our studies revealed that the utilization of coprogen- and ferrichrome-type siderophores by *A. fumigatus* depends exclusively on Sit1 and Sit2 with the exception of low-efficacy use of ferricrocin by an unknown transporter independent of Sit1 or Sit2. In this respect, it is interesting to note that *A. fumigatus* MirB was previously shown to mediate the utilization of ferricrocin when expressed heterologously in *S. cerevisiae* [17]. With respect to ferrichrome-type siderophores, Sit1 and Sit2 showed overlapping as well as unique substrate specificities: utilization of ferrirhodin and ferrirubin depended exclusively on Sit2, use of ferrichrome A depended mainly on Sit1, and utilization of ferrichrome, ferricrocin, and ferrichrysin was mediated by both transporters. Moreover, it was shown previously that ferrichrome-type molecule VL-2397 is transported exclusively by Sit1 as inactivation of Sit1 rendered *A. fumigatus* resistant to this antifungal drug [30]. Ferrichromes are cyclic hexapeptides consisting of three N^5^-acylated N^5^-hydroxyornithine residues (positions R_4_–R_6_), which provide the hydroxamate groups for iron chelation, and three additional amino acids (positions R_1_–R_3_) [6]. Different ferrichrome-type siderophores differ in the acyl groups present in positions R_4_–R_6_ and the amino acid residues present in positions R_1_–R_3_; whereby glycine is present in position R_3_ in most ferrichrome-types with VL-2397 being an exception. Figure 4 compares the different constituents of the ferrichrome-type siderophores used in this study and their utilization. This comparison allows the following conclusions: (i) both Sit1 and Sit2 accept serine and glycine in positions R_1_ and R_2_; (ii) both Sit1 and Sit2 accept acetyl as acyl-group in R_4_–R_6_; (iii) Sit2 but not Sit1 accepts anhydromevalonyl as acyl-group in positions R_4_–R_6_; (iv) Sit2 does not distinguish between *cis-* and *trans-* anhydromevalonyl as acyl group in positions R_4_–R_6_; (v) Sit1, and to a lesser extent Sit2, accept methylglutaconyl as acyl group in positions R_4_–R_6_, (vi) methylglutaconyl as acyl-group in R_4_–R_6_ significantly decreases uptake efficacy in comparison to anhydromevalonyl as seen from the significant differences in growth promotion, and (vii) Sit1 accepts asparagine, leucine, and D-phenylalanine in positions R_1_, R_2_, and R_3_, while at least one of these amino acid residues disturbs recognition by Sit2. These results demonstrate that both the amino acid residues in positions R_1_–R_3_ as well as the acyl-groups impact recognition of ferrichrome-type siderophores by SITs. The data provide structural insights in the substrate specificity of Sit1 and Sit2, which will help to understand substrate recognition. Similar to ferrichrome A, the utilization efficacy of both coprogen-type siderophores tested, coprogen and coprogen B, was low despite the fact that these siderophores were accepted by both Sit1 and Sit2. Neither Sit1 nor Sit2 were important for the utilization of the endogenous siderophores fusarinine C and TAFC, which does not exclude a role in the transport of these siderophores but excludes a dominant role. Moreover, heterologous expression in *S. cerevisiae* suggested that MirB transports TAFC [17,56]. Furthermore, *A. fumigatus* was found to lack utilization of the xenosiderophores basidiochrome, rhodotorulic acid, enterobactin, rhizoferrin, ornibactin and schizokinen–which belong to hydroxamate-, catecholate-, carboxylate- and mixed-class siderophores (Table 1). Moreover, *A. fumigatus* was previously shown to be unable to utilize the mixed-class siderophore pyoverdine–produced by several *Pseudomonas* species [31,57,58]. These data reveal that despite recognizing several hydroxamate-class siderophores, not all members of this siderophore-class are utilized by *A. fumigatus* and that the hydroxamate groups are insufficient for recognition. Furthermore, from the available data, *A. fumigatus* appears to utilize exclusively hydroxamate-type siderophores.

Previously, C-terminally Venus-tagged Sit1 expressed under the control of a xylose-inducible promoter was shown to be localized in the plasma membrane [30]. Here we demonstrate that both N-terminally and C-terminally Venus-tagged Sit1 versions localize to the plasma membrane. Furthermore, the use of the endogenous promoter demonstrated that the expression of *sit1* is repressed by iron at the protein level as previously indicated by transcriptional analysis [15]. Remarkably, particularly N- but also C-terminal Venus-tagging of Sit1 increased susceptibility to VL-2397, an antifungal drug that is exclusively transported by Sit1 [30]. These results indicate that Venus-tagging increases Sit1 activity, possibly by impacting protein stability or plasma membrane retention. Sit1 has 14 predicted transmembrane domains with both the cytoplasmic N- and C-terminus being cytosolic (Figure 2B), which is in agreement with the predicted lack of a signal sequence [44]. In *C. glabrata* Sit1, a Y575A mutation (exchange of tyrosine to alanine) located in the terminal extracellular loop was found to significantly impair ferrichrome utilization [47]. As shown by the multiple alignment analysis in Figure S7, this tyrosine residue is strictly conserved in all analyzed SITs (corresponding to Y532 in *A. fumigatus* Sit1) with exception of the Sit2 clade (Figure 3 and Figure S7 in Supplementary Materials) in which tyrosine is replaced by tryptophan in this position. In *A. fumigatus* Sit1, an N479K mutation located in the 13th transmembrane domain (Figure 2B and Figure S6 in Supplementary Materials) was found to render *A. fumigatus* resistant to VL-2397 [46]. According to topology prediction [44] this mutation results in the formation of an additional transmembrane domain (Figure S6), i.e., a dramatic change of domain organization, which might explain the loss of VL-2397 transport activity.

Phylogenetic analysis indicated that the five SITs of *A. fumigatus* belong to different subclades and that despite overlapping substrate specificities, Sit1 and Sit2 are only distantly related. Sit2 is closer related to MirD and the TAFC transporter MirB than to Sit1 (Figure 3). These results underline once more that phylogenetic analysis is of limited value for the prediction of substrate specificity. Furthermore, two members of the SIT family displayed in the phylogenetic analysis (Figure 3) have been shown to accept non-siderophore substrates; i.e., *S. cerevisiae* Gex2 and *S. pombe* Str3 have been reported to transport glutathione and heme, respectively [59,60]. As previously reported [10], all SIT family members of the *Saccharomycotina* species *S. cerevisiae, C. albicans* and *C. glabrata* cluster closely, which indicates a common origin despite having in part different substrates (Table 3). Notably, the subclade containing *A. fumigatus* Sit1 is the closest related to the “*Saccharomycotina* subclade” again indicating a common origin. Within the *A. fumigatus* Sit1 subclade, *F. graminearum* Sit1 has been shown to transport ferrichrome and ferrioxamine B and *C. neoformans* Sit1 was found to transport ferrioxamine B (Table 3). This overlapping substrate specificity of three SITs from different species within one phylogenetic subclade might indicate that the substrate specificity prediction might be possible to a certain extent within SIT subclades.

The genes encoding Sit1 and Sit2 are expressed during infection as shown in a murine aspergillosis model [61], which is expected because all available data indicate that all iron acquisition systems are coregulated including SITs for both endogenous siderophores and xenosiderophores [3]. In agreement, gallium-labelled desferrioxamine B (ferrioxamine B chelating gallium instead of iron) was recently shown to mediated pre-clinical in vivo imaging of *A. fumigatus* infection by positron emission tomography [28], which underlines the potential value of SITs for diagnosis of fungal infections. In a murine aspergillosis model, Sit1 and Sit2 were found to be dispensable for the virulence of *A. fumigatus* [18], which might limit their value for the import of siderophore-based antifungals as their mutation would most likely result in resistance to these drugs without consequence for the virulence potential. Nevertheless, VL-2397, which is imported exclusively by Sit1 [30], displayed antifungal activity with high efficacy in vitro and in vivo in a murine aspergillosis model [46]. The dispensability of Sit1 and Sit2 for virulence is in agreement with the fact that these SITs appear to be important exclusively for the use of xenosiderophores as *A. fumigatus* lacks the production of coprogen- and ferrioxamine-type siderophores and utilizes ferrichrome-type siderophores only intracellularly [10]. In this respect, it is interesting to note that the siderophore content in soil has been reported to range between 2 to 279 nM with ferrichrome- and ferrioxamine-type siderophores being the most common [62,63]. Therefore, Sit1 and Sit2 most likely evolved to save energy for siderophore biosynthesis and to relieve the intermicrobial competition for iron [7]. The importance of xenosiderophore utilization is reflected by the high evolutionary conservation of Sit1 and Sit2.

## Figures and Tables

**Table 2:** Venus-tagging of Sit1 affects VL-2397. The minimum inhibitory concentration (MIC) of VL-2397 was tested in RPMI medium with 24 h incubation as described previously [30].

Strain	MIC [mg/L]
AfS77	1
*sit1* ^N-Venus^	0.125
*sit1* ^C-Venus^	0.5
∆*sit1*	>16

**Table 3:** Previously identified ferrichrome and ferrioxamine transporting SITs including identified substrate specificities.

Species (Number of SITs)	SIT Name ^1^	Substrate ^2^	Non-Substrate ^2^	Reference
*S. cerevisiae* (4)	Arn1 *	ferrichrome, coprogen	ferrioxamine B, VL2397, TAFC, enterobactin	[14,30]
	Sit1/Arn3 *	ferrichrome, coprogen, ferrioxamine B	VL-2397 TAFC enterobactin	[14,30]
*C. albicans* (1)	Sit1/Arn3 *	ferrichrome, ferricrocin, ferrichrysin, ferrirubin, coprogen, TAFC	ferrioxamine B, ferrioxamine E, VL-2397, enterobactin, rhodotorulic acid	[30,48]
*C. glabrata* (1)	Sit1 *	ferrichrome, ferrirubin, coprogen, VL-2397 ^3^	ferrioxamine B, TAFC, enterobactin	[30,47]
*S. pombe* (2)	Str1	ferrichrome	ferrioxamine B	[49,50]
	Str2	ferrichrome, ferrioxamine B		[49]
*Fusarium graminearum* (9)	Sit1	ferrichrome, ferrioxamine B	TAFC	[51]
*Cryptococcus neoformans* (7)	Sit1	ferrioxamine B		[52]
*A. fumigatus* (5)	Sit1	Ferrichrome ferrioxamine B		[18]
	Sit2	ferrichrome		[18]

^1^ SITs with substrate identified by growth assay in the original organism are marked with *; ^2^ ferrichrome-type siderophores are in red, ferrioxamine-type siderophores are in green, coprogen-type siderophores are in blue, TAFC is in orange; VL-2397 is in purple, and other siderophores are in black; ^3^ VL-2397data are deduced from [30].

## Data Availability

Not applicable.

## Supplementary Materials

The following are available online at https://www.mdpi.com/article/10.3390/jof7090768/s1, Figure S1: Deletion scheme of *sit1* and *sit2* genes in *A. fumigatus*, Figure S2: N-terminal and C-terminal Venus-tagging scheme of *sit1* in *A. fumigatus*, Figure S3: Genomic organization of the *fcyB* locus in AfS77 (wild-type) and reconstituted *sit1* and *sit2* strains, Figure S4: Sit1 mediates uptake of acetylated (Acetyl-FoxB) and succinylated (Succinyl-FoxB) ferrioxamine B derivatives, Figure S5: Complementation of Sit1 and Sit2, Figure S6: Schematic illustration of membrane topology of Sit1 with the N479K mutation according to Protter [44], Figure S7: Multiple alignments of SITs for phylogenetic analysis shown in Figure 3, Table S1: *A. fumigatus* strains used in this study, Table S2: Primers used for strains generation, Table S3: Primers used for the generation of digoxigenin-labelled probes for Southern blot analysis.
