# Supplementary material for: The Siderophore Transporters Sit1 and Sit2 Are Essential for Utilization of Ferrichrome-, Ferrioxamine- and Coprogen-Type Siderophores in Aspergillus fumigatus
Source: J Fungi (Basel). 2021 Sep 16;7(9):768. doi: 10.3390/jof7090768 (PMC8470733; doi:10.3390/jof7090768)
Supplement: Supplementary file 1 [file jof-07-00768-s001.zip › jof-1375482-supplementary.pdf]

Journal of Fungi

Supplementary Material

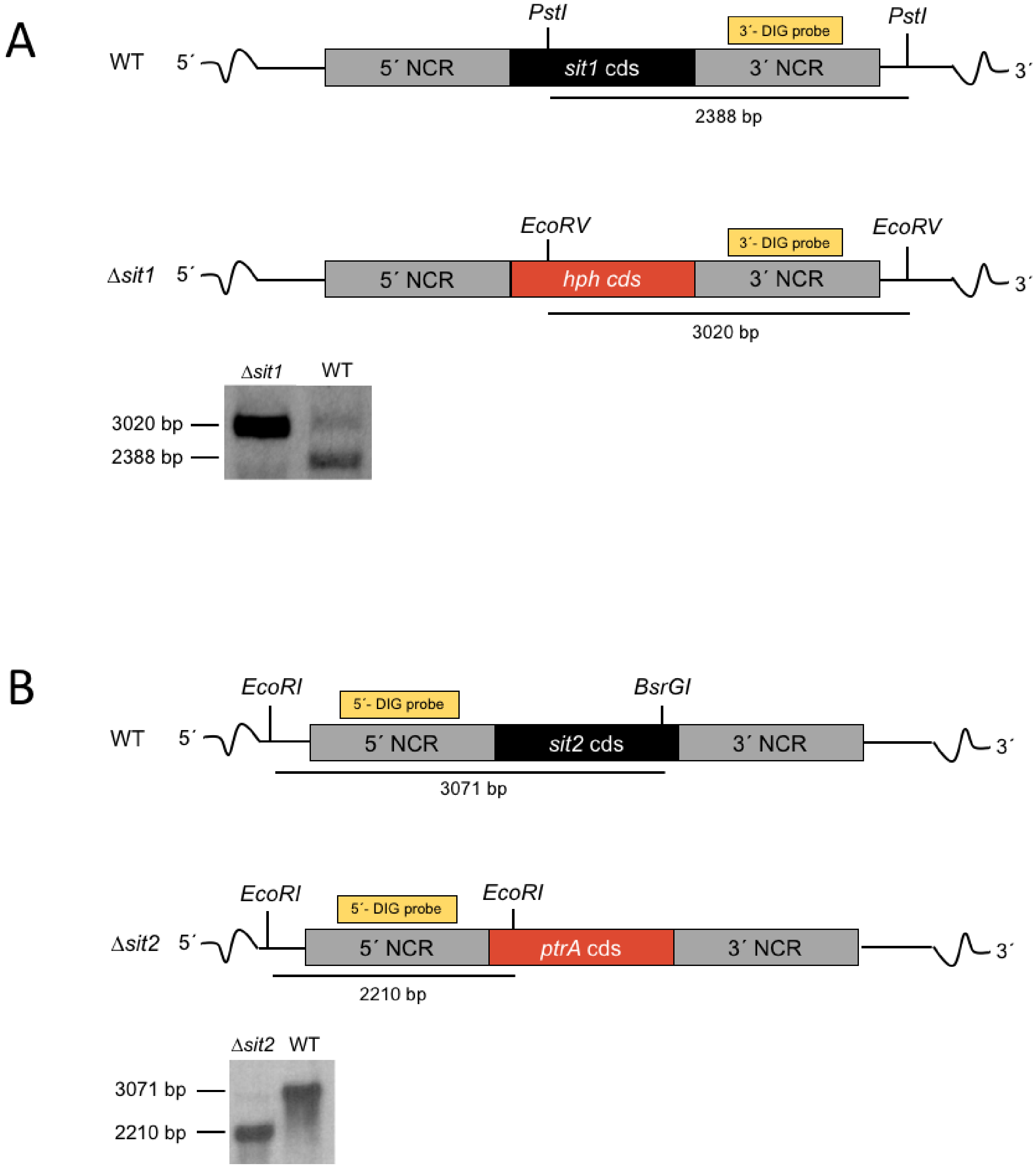

Figure S1. Deletion scheme of *sit1* and *sit2* genes in *A. fumigatus*. **(A)** Genomic organization of the *sit1* locus in AfS77 (wild-type) and Δ*sit1*. DNA digestion with *PstI* resulted in a 2388-bp fragment for AfS77 and digestion with *EcoRV* resulted in a 3020-bp fragment for the Δ*sit1*. **(B)** Genomic organization of the *sit2* locus in AfS77 and Δ*sit2*. DNA digestion with *EcoRI* and *BsrGI*, resulted in a 3071-bp fragment for AfS77 and digestion with *EcoRI* resulted in a 2210-bp fragment for Δ*sit2*. Southern blot analysis using respective DIG hybridization probes confirmed genetic manipulation.

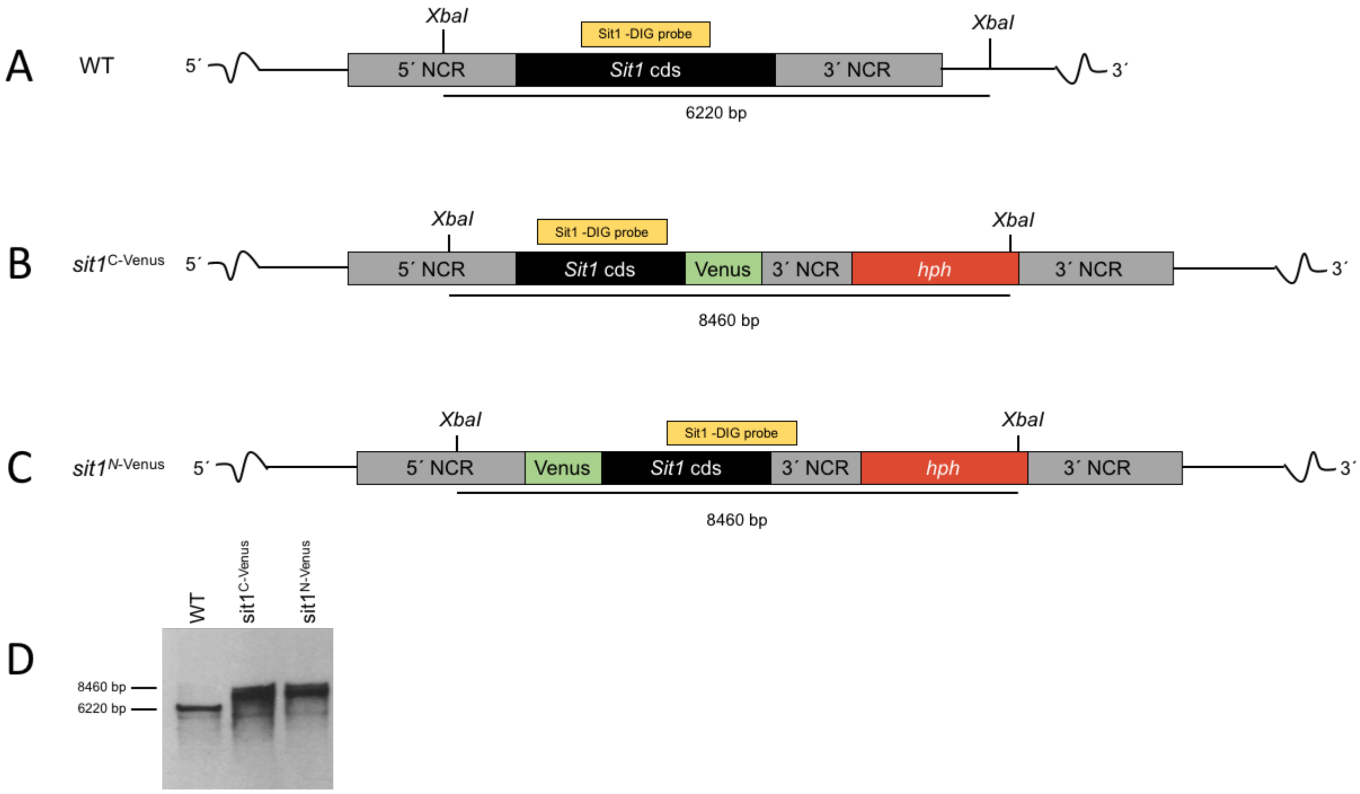

Figure S2. N-terminal and C-terminal Venus-tagging scheme of *sit1* in *A. fumigatus.* **(A)** Genomic organization of the *sit1* locus in AfS77 (wild-type), **(B)** Sit1-Venus at the C-terminus, **(C)** Sit1-Venus at the N-terminus. **(D)** Southern blot analysis using respective DIG hybridization probes confirmed genetic manipulation of strains. DNA digestion with *XbaI* resulted in a 6220-bp fragment for AfS77 and in an 8460-bp fragment for tagging of Sit1 with Venus.

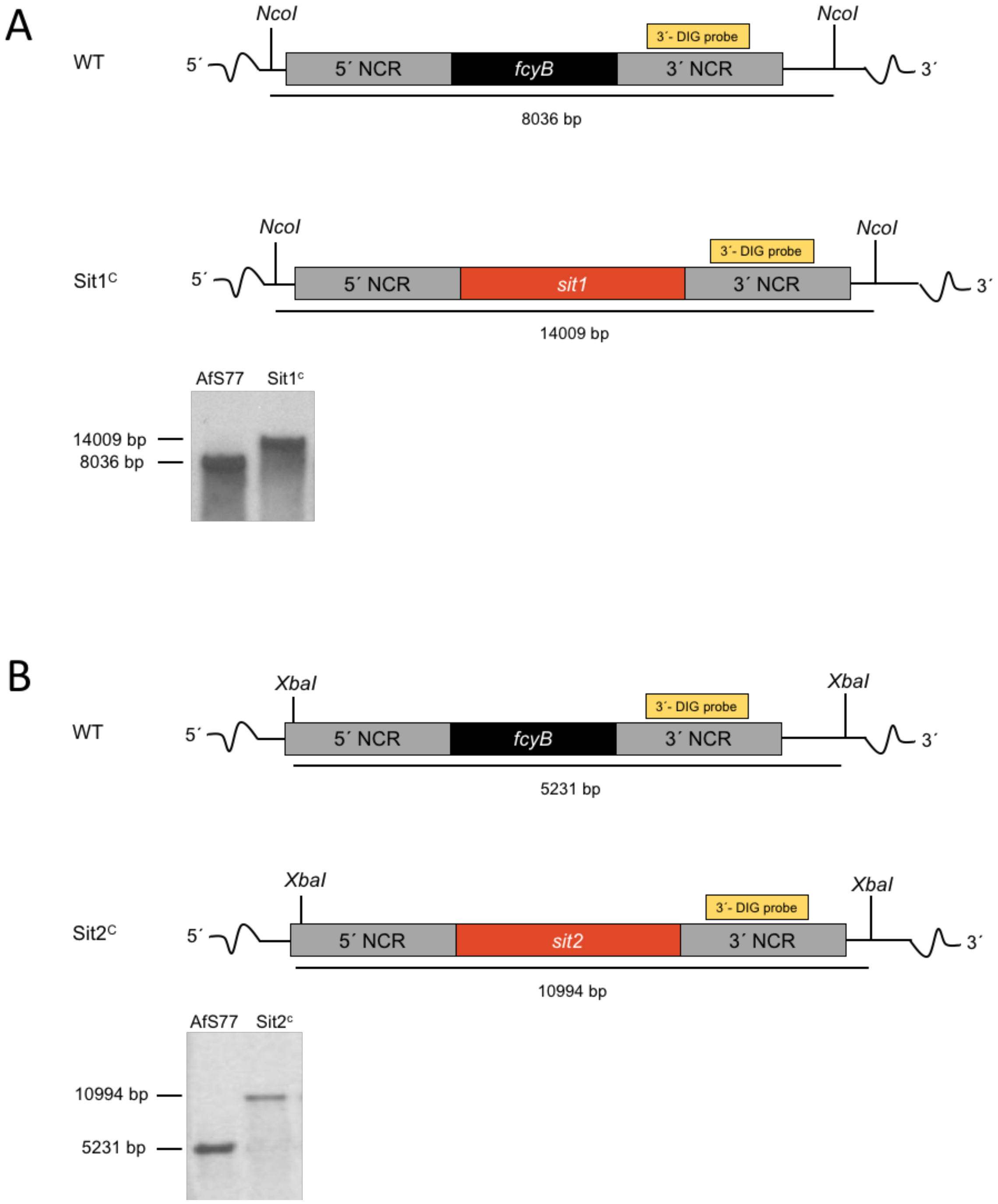

Figure S3. Genomic organization of the *fcyB* locus in AfS77 (wild-type) and reconstituted *sit1* and *sit2* strains. **(A)** DNA digestion with *NcoI* resulted in an 8036-bp fragment for AfS77 and a 14009-bp fragment for complemented Sit1^{C} strain. **(B)** DNA digestion with *XbaI* resulted in a 5231-bp fragment for AfS77 and a 10994-bp fragment for complemented Sit2^{C} strain.

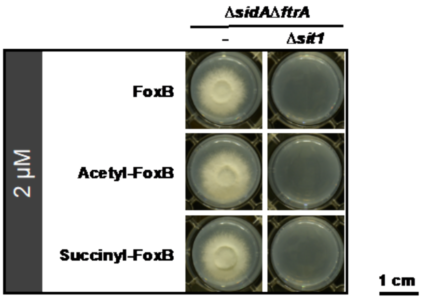

Figure S4. Sit1 mediates uptake of acetylated (Acetyl-FoxB) and succinylated (Succinyl-FoxB) ferrioxamine B derivatives. Utilization of the previously described [28] chemically modified FoxB derivatives was performed as described in Figure 1.

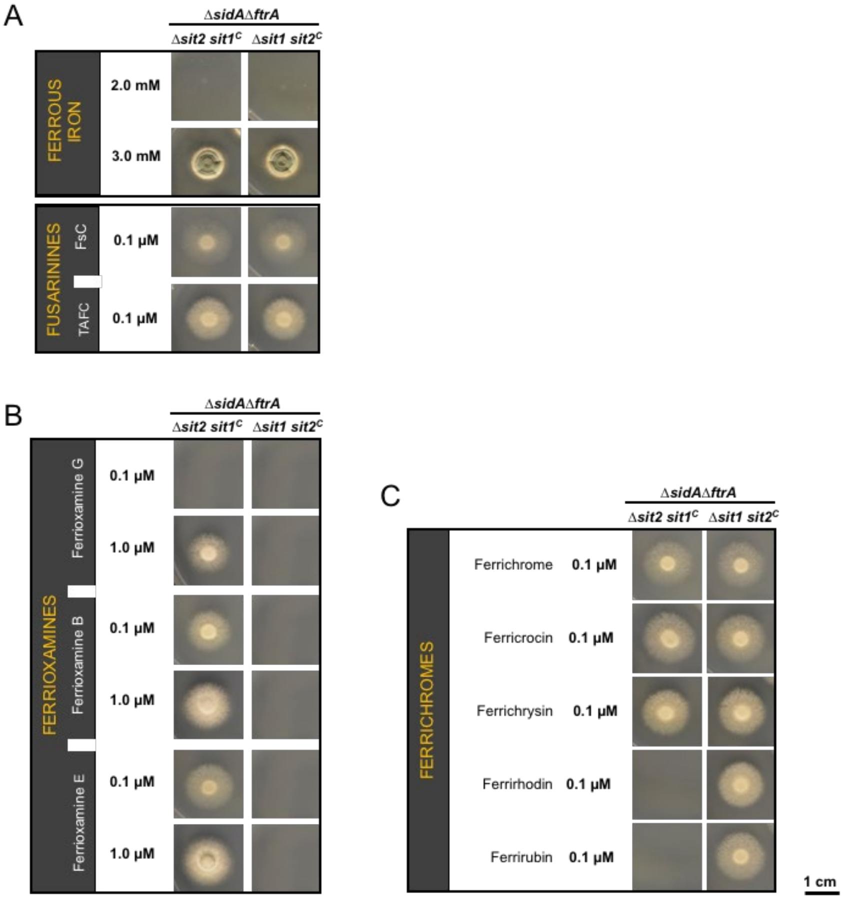

Figure S5. Complementation of Sit1 and Sit2. Strains were point-inoculated with 10^4 conidia on AMM plates supplemented with the indicated siderophore and incubated for 48 h at 37 °C. **(A)** Complemented strains showed growth on 3 mM of Fe^{2+} as well as TAFC and fusarinine C as expected **(B)** Complementation of Sit1 rescued the growth under supplementation of ferrioxamines B, E or G, while for Sit2 no growth is seen still due to the lack of Sit1 transporter. **(C)** Complementation of both Sit1 and Sit2 rescued growth under the supplementation of ferrichrome, ferricrocin and ferrichrysin supporting the role of both transporters in the uptake of these siderophores. Also, the complementation of Sit2 allowed growth under ferrirhodin and ferrirubin but not when only Sit1 was complemented, further demonstrating that Sit2 is the sole transporter for these siderophores.

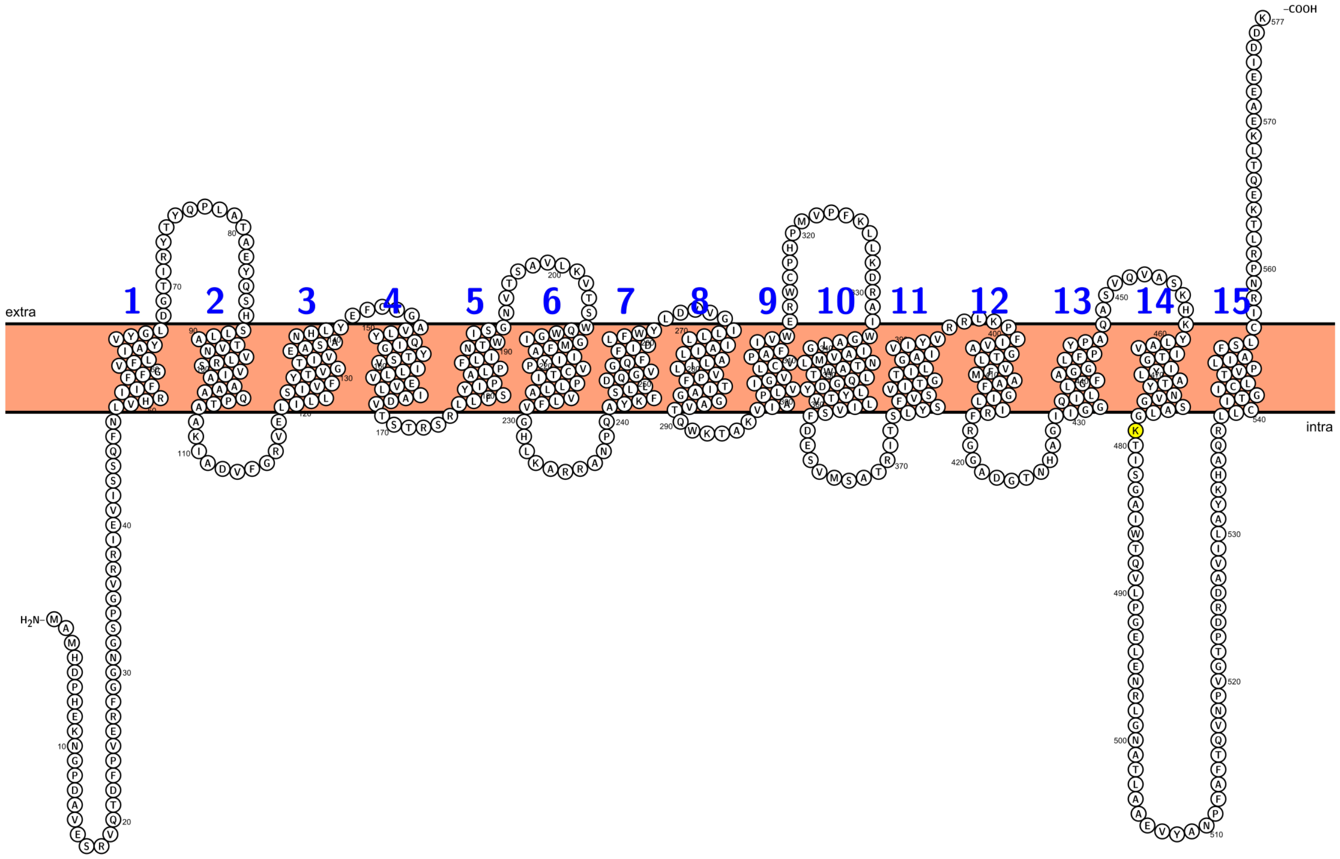

Figure S6. Schematic illustration of membrane topology of Sit1 with the N479K mutation according to Protter [45]. The amino acid residue change N479K (highlighted in yellow), which was found to render *A. fumigatus* resistant to VL-2397 [46], is predicted to lead to a rearrangement of the transmembrane domains in Sit1 (compare to Figure 2B).

Journal of
Fungi

Figure S7. Multiple alignment of SITs for phylogenetic analysis shown in Figure 3. The predicated protein sequence for each transporter was aligned using the Geneious Prime alignment tool [43].

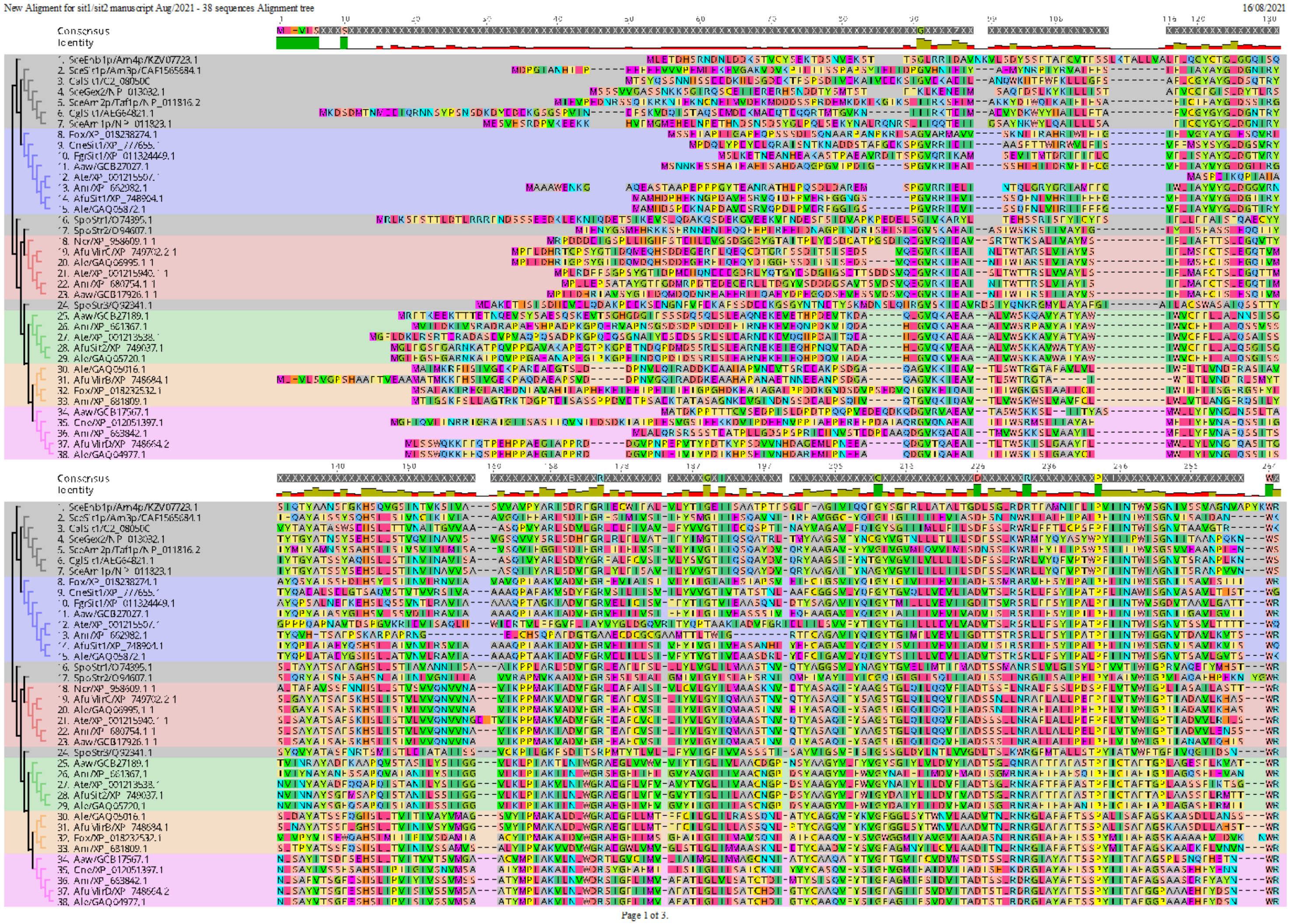

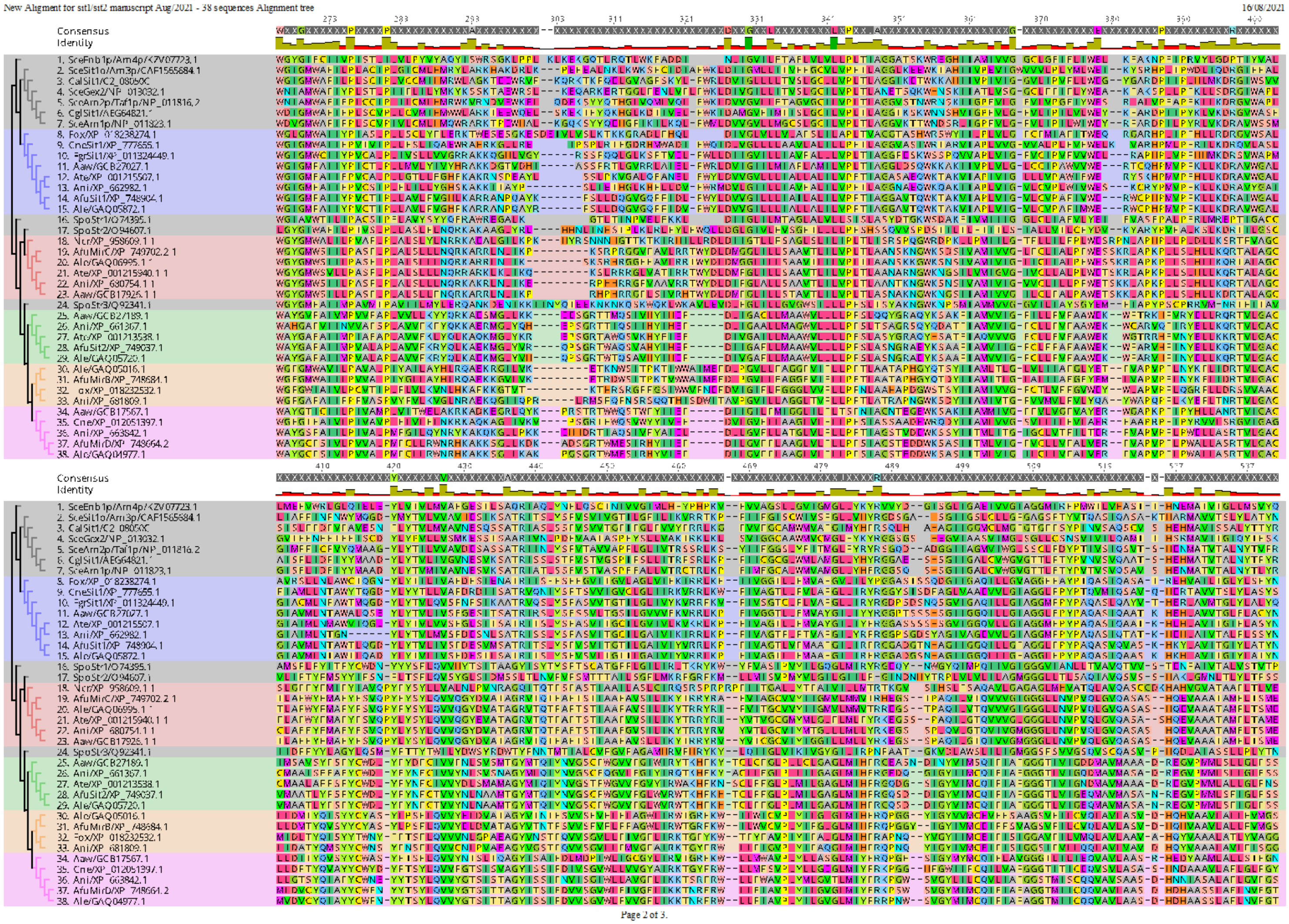
New Alignment for sit1/sit2 manuscript Aug/2021 - 38 sequences Alignment tree
16/08/2021
Consensus
Identity
Consensus
Identity
Page 2 of 3.

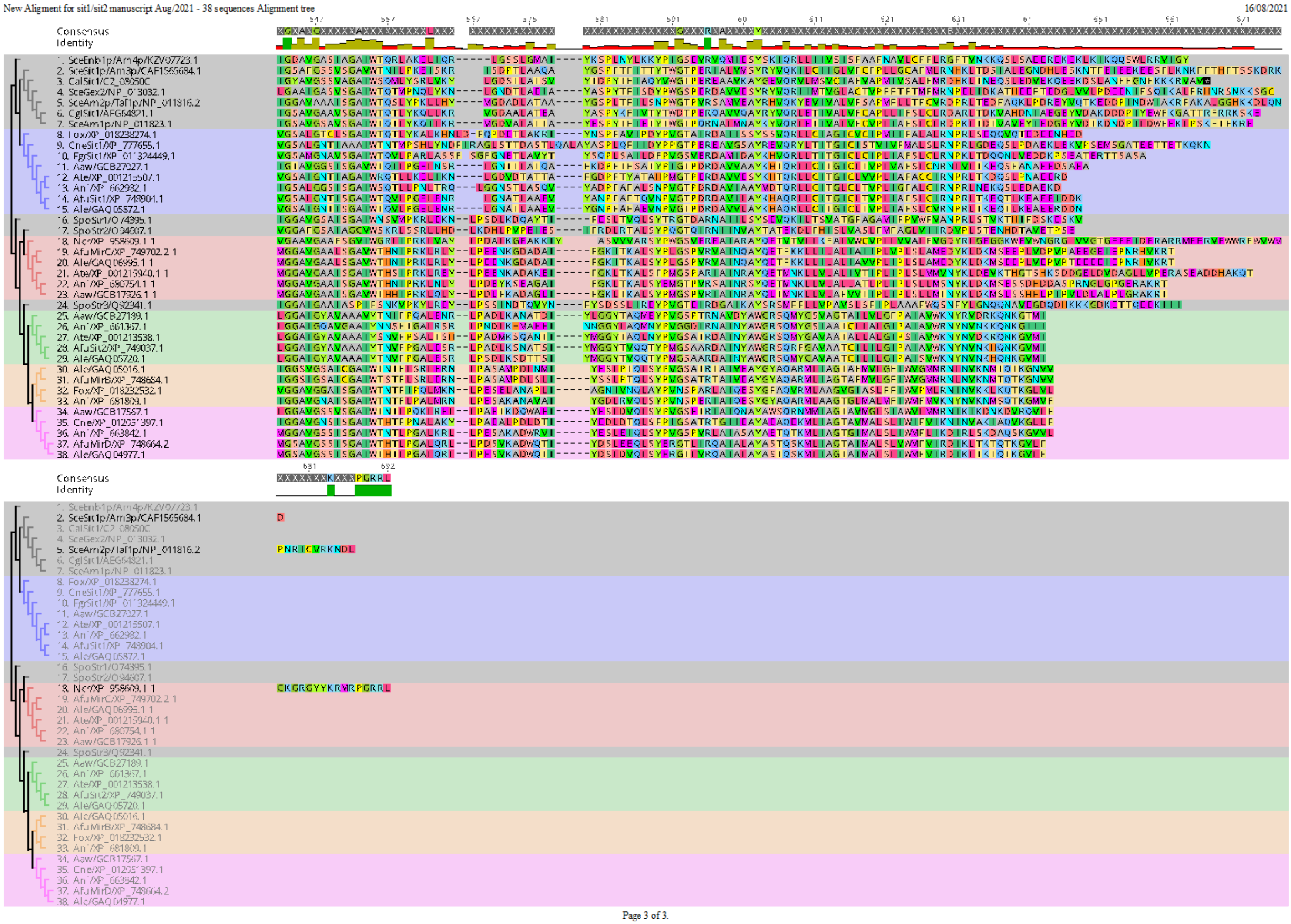
New Aligment for sit1/sit2 manuscript Aug/2021 - 38 sequences Alignment tree
16/08/2021
Consensus
Identity
1. SceEnb1p/Arn4p/KZV07723.1
2. SceSit1p/Arn3p/CAF1565684.1
3. CalSit1/C2_08050C
4. SceGex2/NP_013032.1
5. SceArn2p/Taf1p/NP_011816.2
6. CglSit1/AEG64821.1
7. SceArn1p/NP_011823.1
8. Fox/XP_018238274.1
9. CneSit1/XP_777655.1
10. FgrSit1/XP_011324449.1
11. Aaw/GCB27027.1
12. Ate/XP_001215507.1
13. An/XP_662982.1
14. AfuSit1/XP_748904.1
15. Ale/GAQ05872.1
16. SpoStr1/O74395.1
17. SpoStr2/O94607.1
18. Ncr/XP_958609.1
19. AfuMirC/XP_749702.2
20. Ale/GAQ06995.1
21. Ate/XP_001215940.1
22. An/XP_680754.1
23. Aaw/GCB17926.1
24. SpoStr3/Q92341.1
25. Aaw/GCB27189.1
26. An/XP_661367.1
27. Ate/XP_001213538.1
28. AfuSit2/XP_749037.1
29. Ale/GAQ05720.1
30. Alc/GAQ05016.1
31. AfuMirB/XP_748684.1
32. Fox/XP_018232532.1
33. An/XP_681809.1
34. Aaw/GCB17567.1
35. Cne/XP_012051397.1
36. An/XP_663842.1
37. AfuMirD/XP_748664.2
38. Ale/GAQ04977.1
Page 3 of 3.

Table S1. *A. fumigatus* strains used in this study.

Strain	Description	Reference
AfS77	ATCC4664; Δ*akuA*::*loxP*	[33]
sit1$^{\text{N-Venus}}$	AfS77; *sit1*::*hph*-p*sit1*-Venus^{N}	this study
sit1$^{\text{C-Venus}}$	AfS77; *sit1*::*hph*-p*sit1*-Venus^{C}	this study
Δ*sit1*	AfS77; Δ*sit1*::*hph*	[30]
Δ*sidA*Δ*ftrA*	AfS77; Δ*sidA*::*six*, Δ*ftr*A::*six*	this study
Δ*sidA*Δ*ftrA*Δ*sit1*	Δ*sidA*Δ*ftrA*; Δ*sit1*::*hph*	this study
Δ*sidA*Δ*ftrA*Δ*sit2*	Δ*sidA*Δ*ftrA*; Δ*sit2*::*ptrA*	this study
Δ*sidA*Δ*ftrA*Δ*sit1*Δ*sit2*	Δ*sidA*Δ*ftrA*; Δ*sit1*::*hph*, Δ*sit2*::*ptrA*	this study
Δ*sidA*Δ*ftrA*Δ*sit2sit1*^{C}	Δ*sidA*Δ*ftrA*; Δ*sit1*::*hph*, Δ*sit2*::*ptrA*, Δ*fcyB*::*sit1*	this study
Δ*sidA*Δ*ftrA*Δ*si12sit2*^{C}	Δ*sidA*Δ*ftrA*; Δ*sit1*::*hph*, Δ*sit2*::*ptrA*, Δ*fcyB*::*sit2*	this study

Copyright: © 2021 by the authors. Licensee MDPI, Basel, Switzerland. This article is an open access article distributed under the terms and conditions of the Creative Commons Attribution (CC BY) license (http://creativecommons.org/licenses/by/4.0/).

Table S2. Primers used for strains generation.

Primer	Sequence 5′–3′
MM124	GGCATGCAAGCTTGGCGT
MM125	GTACCGAGCTCGAATTCACTG
TO16	AATTCGAGCTCGGTACTGCGCACAAAA GAGGACGAGCCAC
TO17	AGGACCTGAGTGATGCTCTGACAACAC GATTGGAACTCC
TO18	ATGGTCCATCTAGTGCTTCCAGGTGGA AGCAAGTCAGG
TO19	GCCAAGCTTGCATGCCTGCGCACACTGCTTCTGACTATCATGC
TO20	AATTCGAGCTCGGTACTTTAAAGACGA TGAACACGAATTGAGAGG
TO21	AGGACCTGAGTGATGCCTTGTGAGTCG CGAGGGAGACG
TO22	ATGGTCCATCTAGTGCACGAGTGACCC CCAAAGAGG
TO23	GCCAAGCTTGCATGCCTTTAAATATGA CGACCTTGGTCCATG
TO56	TGCGCACAAAAGAGGACG
TO57	CACACTGCTTCTGACTATC
TO60	ATGAACACGAATTGAGAGG
TO61	TGACGACCTTGGTCCATG
TO102	AAGCTCGTCCCCTCCAG
TO105	GCTCGGTCAGAAAGTCG
MA01	AATTCGAGCTCGGTACCTCCGTTGTCCAGGGTCAGTACAG
MA02	AATCAATTGCTGATGTATATTATCCTCCTTCC
MA03	ACATCAGCAATTGATTACGGATCCCATTGGT
MA04	TATCTCCCTCTTGCATCTTTGTTTGTATTATA
MA05	ATGCAAGAGGGAGATAATTCTAAAGTATATGT
MA06	GCCAAGCTTGCATGCCGGTTGGCCTGCAACGAGGCTTGTC
MA07	AGTGAATTCGAGCTCGGTACAAGCTCGTCCCCTCCAGC
MA12	TGTACCTAGGCTCTGATGGCGAATACGATCTTTTC
MA13	GCCATCAGAGCCTAGGTACAGAAGTCCAATTG

MA14	GATTAGTATATCTAGAAAGAAGGATTACCTC
MA15	TCTTTCTAGATATACTAATCTTCTAAAAATAACGC
MA16	TTACGCCAAGCTTGCATGCCGATACATATTCGTATCTTATGTCTG
MA17	TGCTGACCATTCGCAGATCGTCACTGGTATAG
MA18	CGATCTGCGAATGGTCAGCAAGGGCGAG
MA19	CCGGACCCGGACCCCTTGTACAGCTCGTCCATGC
MA20	GCTGTACAAGGGGTCCGGGTCCGGGTCCATGAACATGGCGATGCAC
MA53	AATCATGGTCATAGCTGTTTGCTGGAGCAATGGGACGG
MA54	GAGCGGATAACAATTTCACATCTGGATTTTTGCCGACTTTGT
MA55	AATCATGGTCATAGCTGTTTCGACCCATAAAGCGTCATCAG
MA56	GAGCGGATAACAATTTCACACAGAGGACTGAGCTCCGATC

Table S3. Primers used for the generation of digoxigenin-labelled probes for Southern analysis.

Probe	Gene	Sequence 5´–3´
3′ NCR *sit1*	AFUA_7G06060<br>siderophore transporter	AAGCTCGTCCCCTCCAG<br>CCATTAGTGGTGGGGTTC
sit1-CDS	AFUA_7G06060<br>siderophore transporter	AGAACCAACCATGAACATGGCGATGCAC<br>TTACGGATGATTTATCATCAATCTCCTCCG
5′ NCR sit2	AFUA_7G04730<br>siderophore transporter	CATGCTCGAGAAACCAATG<br>TGGAGGAGGAGAAGAGTG
3′ NCR fcyB<br>(for *sit1*^{C} and *sit2*^{C})	AFUA_2G09860<br>cytosine transporter	GCTCTGAACGATATGCTCCCTGCGGTTTTTGGGTTTTAT<br>CACACTGGGTCTGAAGACGA
